# Differential Dynamic Reorganization of Functional Connectivity Based on Phase Synchrony and Amplitude Envelope Coupling During Propofol Sedation

**DOI:** 10.3390/brainsci16080866

**Published:** 2026-08-16

**Authors:** Zhilei Lan, Xiaoli Li, He Chen

**Affiliations:** 1 School of Automation Science and Engineering, South China University of Technology, Guangzhou 510641, China; lzl8031@163.com (Z.L.); xiaolili@scut.edu.cn (X.L.); 2 State Key Laboratory of Cognitive Neuroscience and Learning, Beijing Normal University, Beijing 100875, China

**Keywords:** electroencephalography, propofol, consciousness, dynamic functional connectivity, weighted Phase Lag Index, amplitude envelope correlation

## Abstract

**Highlights:**

**What are the main findings?**

- During propofol sedation, phase synchronization and amplitude envelope coupling in the alpha band exhibit spatially differential reorganization, suggesting that they reflect distinct neural synchronization mechanisms.
- The occurrence rates of dynamic functional connectivity states derived from phase synchronization and amplitude envelope coupling change selectively with deepening sedation, and these changes are closely associated with individual behavioral responsiveness levels.

**What are the implications of the main findings?**

- The findings challenge the simplified view that the level of consciousness declines along a single dimension with increasing anesthesia depth, suggesting instead that conscious states may depend on the synergistic maintenance of multiple neural synchronization mechanisms.
- This study provides a basis for developing novel EEG-based dynamic network state markers for monitoring depth of anesthesia and provides new ideas for investigating the network reorganization patterns underlying fluctuations in consciousness.

**Abstract:**

Background/Objectives: Consciousness fluctuations involve brain network reorganization, yet the underlying neural synchronization mechanisms remain unclear. This study examined the static and dynamic characteristics of alpha-band functional connectivity during propofol sedation from two dimensions: phase synchrony and amplitude coupling. Methods: Electroencephalography data from 20 healthy volunteers across baseline, mild sedation, moderate sedation, and recovery were analyzed. Source-level signals for 68 cortical regions of interest were reconstructed using sLORETA. Dynamic functional connectivity matrices for both weighted Phase Lag Index (wPLI) and amplitude envelope correlation (AEC) were computed using 5 s sliding windows. Dynamic connectivity states were identified through clustering analysis, and state occurrence rates were compared between drowsy and responsive participants across sedation levels. Results: Static analysis revealed a dissociation between the two metrics: during moderate sedation, wPLI showed significant suppression in posterior parieto-occipital regions, whereas AEC exhibited widespread whole-brain coupling enhancement. Dynamic clustering identified three wPLI states and five AEC states. Critically, although the two metrics exhibited spatially distinct dynamic reconfiguration patterns, with deepening sedation, the occurrence rate of the ventral connectivity pattern in wPLI and that of the medial prefrontal pattern in AEC both increased significantly, and these two patterns showed synergistic co-occurrence. This effect was more pronounced in the drowsy subgroup, with greater increases in both patterns. Conclusions: Propofol-induced alterations in consciousness are not characterized by linear attenuation along a single neural synchrony dimension, but rather by differential reorganization of phase- and amplitude-based functional connectivity across spatial configurations and temporal dynamics.

## 1. Introduction

The dynamic fluctuation of consciousness levels and its underlying neural mechanisms represent a core issue of common concern in both cognitive neuroscience and clinical medicine [1,2]. General anesthetics, by reversibly modulating central nervous system activity, provide an ideal experimental model for investigating transitions in consciousness states [3]. Propofol, the most used intravenous anesthetic in clinical practice, acts primarily by enhancing γ-aminobutyric acid (GABA)-mediated inhibitory neurotransmission [4] and can reliably induce a continuous spectrum of state changes from wakefulness and sedation to loss of consciousness [5,6]. This pharmacological model enables researchers to examine the brain network reorganization accompanying consciousness fluctuations under well-controlled experimental conditions [7,8,9].

Resting-state electroencephalography (EEG), with its millisecond-level temporal resolution, offers unique advantages in capturing the rapid neural dynamics during consciousness fluctuations. A substantial body of evidence has established that alpha-band (8–13 Hz) cortical oscillations play a critical role in propofol sedation [7,10]. Propofol induces frontal dominance of alpha power and drives a spatial shift of alpha oscillations from occipital to frontal regions [11,12,13]. However, the dynamic changes in alpha-band functional connectivity during sedation, particularly whether phase synchrony and amplitude coupling follow the same reorganization principles, remain insufficiently understood.

Functional connectivity can typically be characterized from two complementary perspectives [14]. The weighted Phase Lag Index (wPLI) quantifies the consistency of phase difference distributions between brain regions, reflecting precise temporal synchronization of neural activity and serving as a foundation for long-range communication and information integration [15]. Amplitude Envelope Correlation (AEC), in contrast, measures the coupling of amplitude envelopes between brain regions, reflecting slower fluctuations in population-level excitability [16,17]. These two metrics capture distinct facets of neural synchronization and may be governed by different neural regulatory mechanisms [14,18].

Previous investigations examining the effects of propofol on functional connectivity have predominantly treated functional connectivity as a unitary construct or focused exclusively on differences in its static averages across experimental conditions [19]. These studies typically report widespread reductions in functional connectivity under sedation, yet they rarely differentiate the distinct contributions of phase synchrony versus amplitude coupling or examine the temporal reorganization of connectivity patterns [7,8]. Resting-state brain networks are not static entities but rather continuously switch among multiple spatial configurations [20]. Fluctuations in consciousness levels are likely accompanied by alterations in this dynamic switching behavior, which static averaging approaches are inherently ill-suited to capture [21,22].

The present study aimed to systematically characterize the changes in alpha-band phase synchrony and amplitude coupling during propofol sedation from static to dynamic levels. Specifically, we sought to address the following questions: First, what are the characteristics of static connectivity patterns for phase synchrony and amplitude coupling across different sedation stages? Second, from a dynamic perspective, how do recurrent brain network connectivity states vary with sedation depth, and do different connectivity metrics capture distinct patterns of dynamic transition? Third, how do the occurrence rates of these network states relate to behavioral measures of responsiveness and drowsiness? We hypothesized that during propofol sedation, alpha-band phase synchrony and amplitude coupling would exhibit different dynamic characteristics, implying that their temporal evolution patterns and spatial reconfiguration features might differ, potentially reflecting distinct underlying neural mechanisms. This differential reorganization may constitute a multidimensional network signature of consciousness states, providing a richer characterization of brain network changes than single-dimensional connectivity measures, and may offer new insights into the mechanisms underlying anesthetic-induced consciousness alterations.

## 2. Materials and Methods

### 2.1. Experimental Design

The data for this study were obtained from a previously published propofol sedation study [23]. The study was approved by the Cambridgeshire 2 Regional Ethics Committee, and all participants provided written informed consent. The research procedures were conducted in accordance with the Declaration of Helsinki. A total of 20 healthy volunteers (9 males, 11 females; mean ± SD age, 30.85 ± 10.98 years) were enrolled.

**Figure 1:**
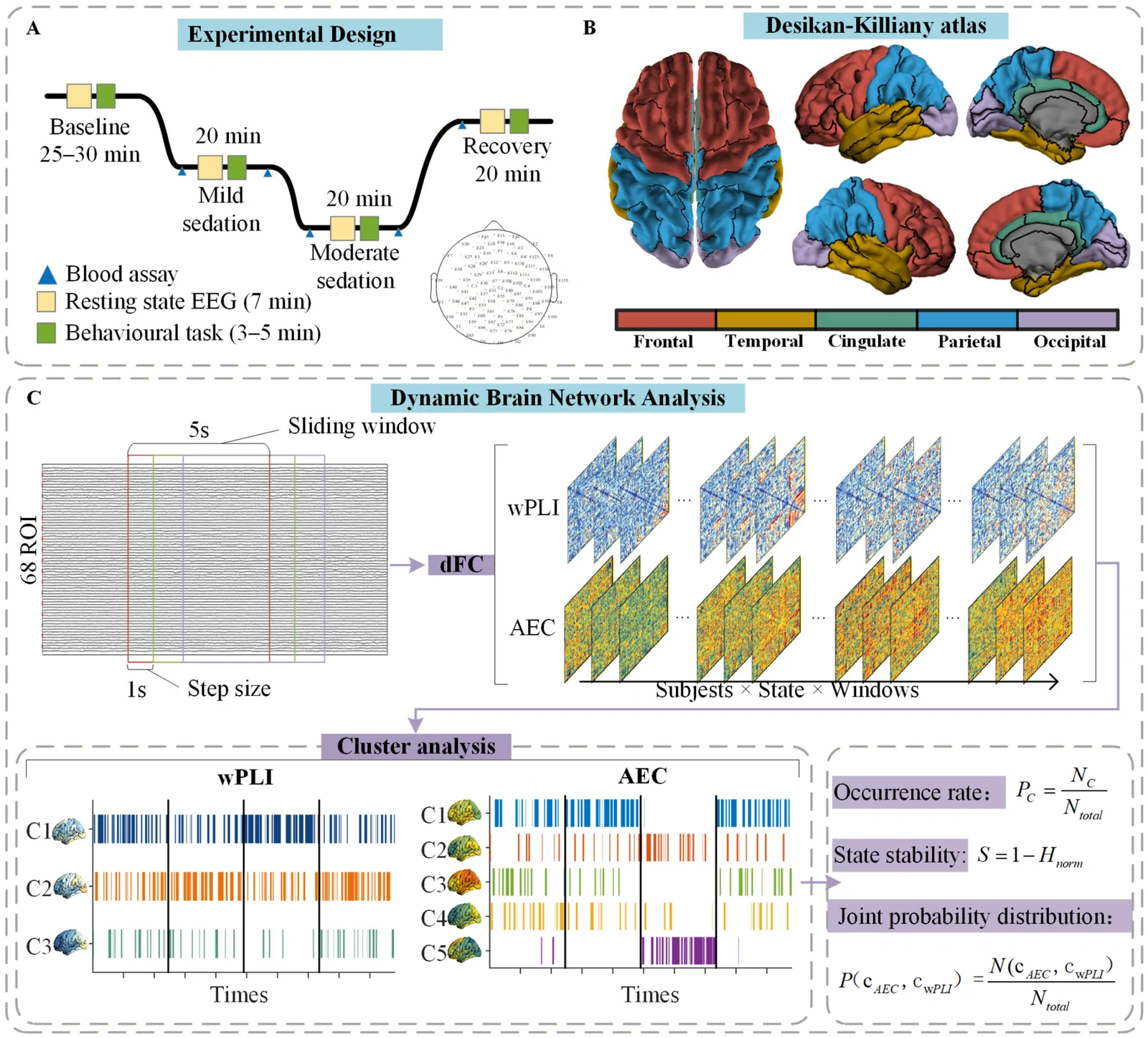
Schematic illustration of the experimental design and dynamic brain network analysis pipeline: (**A**) Four sessions of approximately 7 min resting-state EEG recordings (128 channels) were acquired from each participant, corresponding to baseline (before propofol administration), mild sedation, moderate sedation, and recovery. After each resting-state recording, participants completed a two-choice rapid response task to assess behavioral responsiveness, and blood samples were collected. (**B**) Source-space analysis parcellated 68 regions of interest into five major brain regions based on the Desikan–Killiany atlas: frontal (red), temporal (yellow), cingulate (green), parietal (blue), and occipital (purple). (**C**) Dynamic functional connectivity was constructed by computing wPLI and AEC using sliding time windows, followed by clustering to identify discrete connectivity states and further dynamic analysis.

Propofol was administered using a target-controlled infusion system (Alaris Asena PK, Carefusion, Basingstoke, UK). The experiment comprised four stages: baseline, mild sedation (target concentration 0.6 μg/mL), moderate sedation (target concentration 1.2 μg/mL), and recovery. At each target level, a 10 min stabilization period was allowed for equilibration of plasma propofol concentrations to attain a steady state before data acquisition. At each stage, approximately 7 min of resting-state EEG were acquired after stabilization of the targeted effect site concentration. At each steady-state level, participants performed an auditory discrimination task (discriminating broadband noise from 150 Hz harmonic complex tones), with behavioral responses assessed by accuracy (hit rate) and reaction time. Based on behavioral performance during moderate sedation, participants were divided into a drowsy group (*n* = 7, exhibiting behavioral impairment) and a responsive group (*n* = 13, maintaining responsiveness with prolonged reaction times). Blood samples were collected at each stage to determine actual plasma propofol concentrations. Figure 1 illustrates the overall experimental design and the subsequent dynamic brain network analysis pipeline.

### 2.2. Data Acquisition and Preprocessing

EEG signals were recorded using a 128-channel system (Net Amps 300, Electrical Geodesics Inc., Eugene, OR 97401, USA) at a sampling rate of 250 Hz, with the vertex electrode used as the reference. A total of 91 scalp electrodes were retained for analysis, while electrodes over the neck, cheeks, and frontal regions were excluded. The signals were band-pass filtered at 0.5–45 Hz, segmented into 10 s epochs, and baseline-corrected using the mean voltage over the entire recording period. A semi-automated procedure (normalized variance calculation combined with visual inspection) was applied to reject epochs and channels contaminated by ocular or myogenic artifacts. After preprocessing, the number of retained epochs (mean ± SD) for each stage was as follows: baseline 38 ± 5, mild sedation 39 ± 4, moderate sedation 38 ± 4, and recovery 40 ± 2. Rejected channels were interpolated using spherical spline interpolation, and the data were re-referenced to the common average reference. The preprocessing described above was implemented using the EEGLAB toolbox [24] in MATLAB (version R2024a).

To reconstruct brain networks at the cortical level and to reduce the effects of volume conduction, source imaging analysis was performed on the preprocessed EEG data [25]. A forward head model was constructed using the OpenMEEG boundary element method [26] based on the ICBM152 default brain template implemented in the Brainstorm toolbox (version 20-Apr-2026) [27]. Subsequently, the sLORETA algorithm [28] was applied to estimate current density at 15,002 cortical voxels, with dipole orientations constrained to be perpendicular to the cortical surface. To obtain region-level time series, these voxels were parcellated into 68 regions of interest (ROIs) according to the Desikan–Killiany brain atlas [29], with the source activity of each ROI represented by the mean current density across all voxels within that region. This atlas covers 34 brain regions in each hemisphere, encompassing the major cortical areas, including the frontal, parietal, temporal, occipital, and cingulate cortices. The specific parcellation of brain regions is detailed in Table 1.

### 2.3. Dynamic Functional Connectivity

#### 2.3.1. Weighted Phase Lag Index

Phase synchrony analysis was performed using the wPLI [15]. The calculation procedure was as follows:

For each ROI, the alpha-band source signal was first transformed via the Hilbert transform to obtain the analytic signal. For any pair of ROIs, *i* and *j*, the imaginary part of their cross-spectrum was computed, and then the wPLI was calculated according to the following formula:

(1) $${\mathrm{wPLI}}_{ij}=\frac{|\langle Im(C_{ij})\rangle |}{\left\langle |Im(C_{ij})|\right\rangle }$$

This yielded the uncorrected wPLI values, where $C_{ij}$ represents the cross-spectrum of the two signals and ⟨⋅⟩ denotes averaging over time. The calculation was performed using a 5 s sliding window with a 1 s step size, resulting in a 68 × 68 connectivity matrix.

To eliminate spurious connections arising from non-phase-synchronized activity, a surrogate data method was employed for statistical correction. Specifically, for the time series of each channel, a random cut point was selected, and the two subsequences before and after this point were swapped to generate shuffled surrogate sequences [30]. For each pair of ROIs, the original Hilbert transform of one channel was retained, while the Hilbert transform of the other channel was replaced with the shuffled version to compute surrogate wPLI values. This procedure was repeated N times (in this study, N=1000) to obtain a distribution of surrogate wPLI values for each ROI pair [31]. For each ROI pair, the measured wPLI value was compared against the surrogate distribution using the Wilcoxon signed-rank test. If p<0.05, the connection was deemed statistically significant, and the corrected wPLI value was obtained by subtracting the surrogate median from the measured wPLI; otherwise, the connection was set to zero. This procedure ultimately yielded a statistically corrected 68 × 68 connectivity matrix.

#### 2.3.2. Amplitude Envelope Correlation

To quantify the synchrony of amplitude fluctuations in source signals, we constructed functional connectivity matrices based on AEC. To attenuate signal leakage caused by volume conduction, pairwise orthogonalization [17] was applied to the alpha-band source signals for each pair of ROIs [32]. Taking an arbitrary pair, let y be the reference and x the signal to be corrected. The linear contribution coefficient of y to x, $\beta =y^{†}x$ (where $y^{†}$ is the pseudoinverse of y), was subtracted from x to obtain $x_{c}=x-y\beta$. The Hilbert transform was then applied to $\left[x_{c},y\right]$ to extract amplitude envelopes, which were demeaned before computing the Pearson correlation coefficient. To avoid directional bias arising from the asymmetric nature of pairwise orthogonalization, bidirectional orthogonalization was performed for all ROI pairs, and the final AEC value was defined as the average of the two correlation coefficients. Temporally, a 5 s sliding window with a 1 s step size was applied, and within each window, all pairwise AEC values across the 68 ROIs were computed to construct a 68 × 68 connectivity matrix.

### 2.4. Clustering Analysis

To reveal the dynamic patterns of brain functional connectivity across different consciousness states, dimensionality reduction and clustering were performed separately for the wPLI and AEC dynamic functional connectivity metrics [33,34]. For each metric, all 68 × 68 connectivity matrices corresponding to all sliding windows across all participants and all states were stacked together, forming a three-dimensional data array containing 19,600 samples (total samples = 20 participants × 4 consciousness states × 245 time windows). For each sample’s 68 × 68 connectivity matrix, the upper triangular elements above the main diagonal (totaling 68 × 67/2 = 2278 connectivity features) were extracted and unfolded into a feature vector. This procedure yielded a sample-by-feature matrix of size 19,600 × 2278, where each row corresponded to a sample and each column to a connectivity edge.

To remove redundancy and suppress noise, principal component analysis (PCA) was first applied for linear dimensionality reduction, retaining principal components that explained 70% of the total variance. Subsequently, based on the PCA results, the nonlinear dimensionality reduction method Sampling-enabled Uniform and Discriminative Embedding (SUDE) was further applied to reduce the features to 15 dimensions [35]. On the low-dimensional embedding representations, k-means clustering was applied (100 repetitions) to ensure stability. The optimal number of clusters was determined by integrating the elbow method, silhouette coefficient, and gap statistic (50 reference datasets, PCA reference distribution), with the additional criterion that the smallest cluster should contain no less than 10% of all samples to avoid excessively fragmented and uninterpretable patterns. Bootstrap stability was assessed (B = 500) using the Adjusted Rand Index (mean ARI > 0.99 for both wPLI and AEC), and the k-means solution was further compared with spectral clustering; spectral clustering produced fragmented clusters with excessively small sample sizes, whereas k-means yielded more balanced partitions and was therefore more suitable for the present dataset. Detailed validation results are provided in Supplementary Figures S2 and S3. The resulting cluster labels reflected the prototypical patterns of dynamic functional connectivity changes across different consciousness states. The resulting cluster labels reflected the prototypical patterns of dynamic functional connectivity changes across different consciousness states.

### 2.5. Dynamic Metrics

Based on the time-varying state sequences obtained from clustering, the following temporal metrics were calculated to characterize dynamic connectivity.

State occurrence rate: For each participant at each consciousness level, the occurrence rate [36] of each cluster state was computed as the number of occurrences of that state divided by the total number of windows at that level:

(2) $$P_{c}=\frac{N_{c}}{N_{\mathrm{total}}}$$

where $N_{c}$ is the number of occurrences of state c, and $N_{\mathrm{total}}$ is the total number of time windows at that consciousness level. The occurrence rate reflects the relative activation frequency of a given connectivity pattern at a specific consciousness level.

State stability: For each participant at each consciousness level, the normalized entropy of the state distribution was first computed, and state stability was then defined as one minus this value:

(3) $$S=1-H_{\mathrm{norm}}=1+\frac{\sum_{c=1}^{k}P_{c}\log P_{c}}{\log k}$$

where k is the total number of clusters. A larger S value indicates that the state distribution is more concentrated on fewer patterns, reflecting a more stable and predictable dynamic process, whereas a smaller value indicates more frequent state switching and richer, more diverse dynamic patterns.

Joint probability distribution: To examine the co-occurrence relationship between AEC and wPLI connectivity dynamics, the cluster labels of the two modalities were cross-tabulated to compute the joint probability distribution at each consciousness level, and the distribution was visualized as follows:

(4) $$P(c_{\mathrm{AEC}},c_{\mathrm{wPLI}})=\frac{N(c_{\mathrm{AEC}},c_{\mathrm{wPLI}})}{N_{\mathrm{total}}}$$

This distribution provides an intuitive visualization of the co-varying patterns of the two types of functional connectivity within the same time windows.

### 2.6. Statistical Analysis

All statistical analyses were performed in the R environment (R version 4.5.3). Linear mixed-effects models were used to model state occurrence rates and state stability separately, fitted using the lmer function from the lme4 package, with model parameter estimates and statistical test results obtained via the lmerTest package. For state occurrence rates, the occurrence rate of each cluster state served as the dependent variable. Fixed effects included stage (four levels: baseline, mild sedation, moderate sedation, and recovery), group (two levels: responsive and drowsy), pattern type, and their three-way interaction. The random effect was participant-specific intercepts. For state stability, the stability index *S* served as the dependent variable. Fixed effects included stage, group, and their interaction, with the same random effect of participant intercepts. After model fitting, Type III analysis of variance was employed to test the significance of each fixed effect and their interactions. When significant interaction effects were detected, simple effects analyses were conducted using the emmeans package, with multiple comparisons corrected using the Tukey HSD method, and Cohen’s *d* was calculated as the effect size. The significance levels for statistical results were marked uniformly as follows: p<0.05 denoted by “*”, p<0.01 denoted by “**”, and p<0.001 denoted by “***”.

## 3. Results

To systematically characterize the changes in alpha-band functional connectivity during propofol sedation, we analyzed wPLI and AEC at both static and dynamic levels. Static analyses compared the mean connection strength of whole-brain functional connectivity across stages, whereas dynamic analyses identified recurrent connectivity patterns via clustering and further examined their stability and occurrence rates.

### 3.1. Comparison of Static Alpha-Band Functional Connectivity Between wPLI and AEC Across Different Stages of Propofol Sedation

We calculated the phase-based wPLI and amplitude-based AEC metrics and compared the differences in whole-brain static functional connectivity in the alpha band across the four stages: baseline, mild sedation, moderate sedation, and recovery. The results revealed markedly distinct patterns between the two metrics.

**Figure 2:**
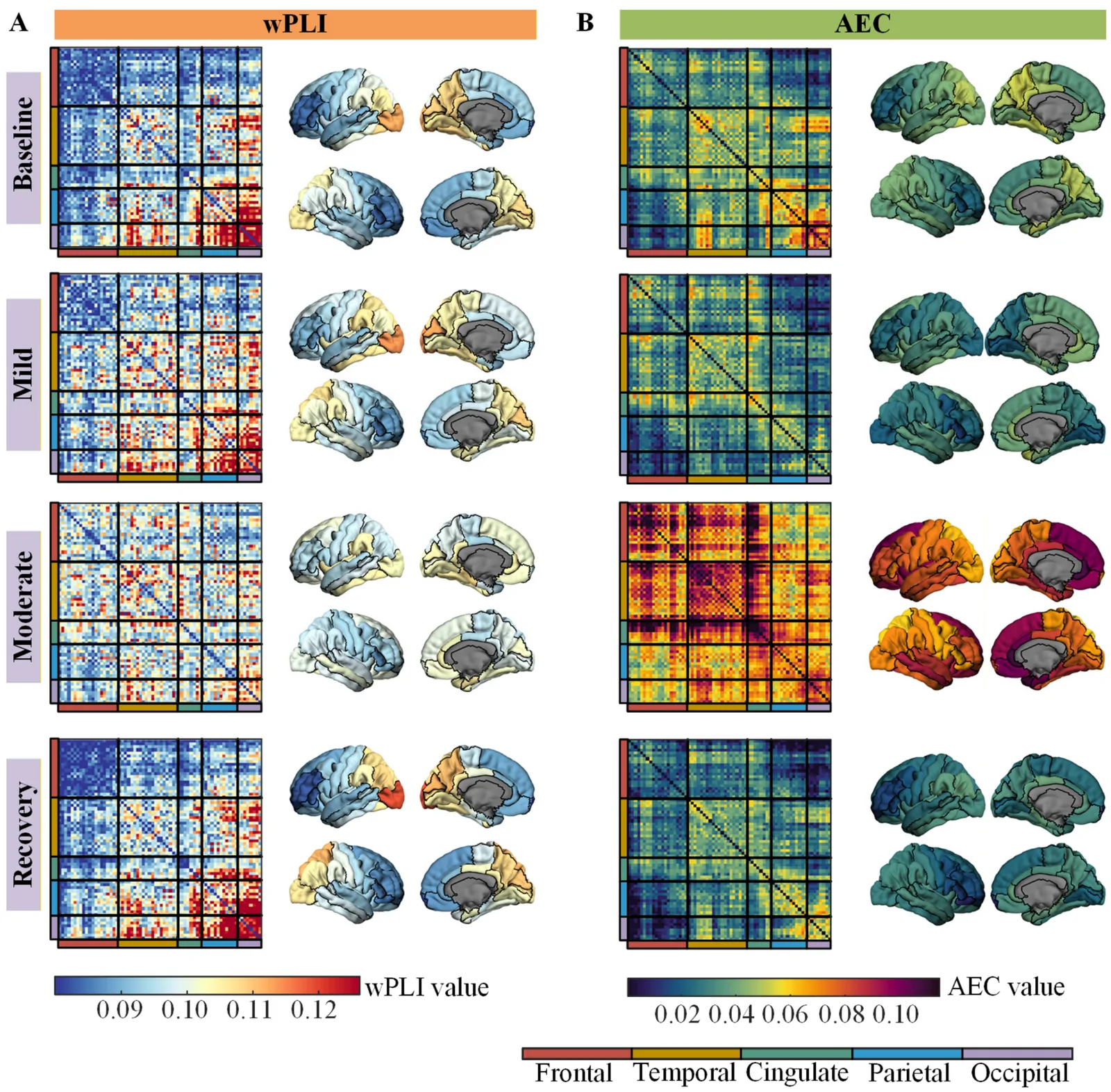
Static functional connectivity results across different consciousness levels: (**A**) Static wPLI functional connectivity. Left panel: group-level wPLI matrices for the four stages (baseline, mild sedation, moderate sedation, and recovery) displayed from top to bottom. Right panel: corresponding cortical ROI projections, with each ROI color representing the mean wPLI connection strength between that node and all other nodes. In the wPLI matrices, cool colors (blue) indicate low connection strength, while warm colors (red) indicate high connection strength. (**B**) Static AEC functional connectivity. Left panel: group-level AEC matrices for the four stages displayed from top to bottom. Right panel: corresponding cortical ROI projections, with each ROI color representing the mean AEC connection strength between that node and all other nodes. AEC values ranged from 0.02 to 0.10. In the AEC matrices, cool colors indicate low connection strength, while warm colors indicate high connection strength. The black horizontal and vertical lines in the matrices demarcate boundaries between different brain regions, with regions ordered from bottom to top (or left to right) as follows: frontal (red), temporal (orange), cingulate (yellow), parietal (blue), and occipital (purple).

At baseline, the wPLI connectivity matrix showed the strongest phase synchrony in the posterior parieto-occipital regions, with the lower-right area of the matrix (parietal–occipital connections) appearing prominently in red, indicating a clear posterior synchrony network (Figure 2A). During mild sedation, this posterior connectivity began to decline. By moderate sedation, posterior synchrony was significantly reduced, with the corresponding matrix regions shifting from red to blue, indicating that the fine-grained phase synchrony in the alpha band was markedly suppressed in the posterior parieto-occipital regions. During recovery, connectivity gradually returned to near-baseline levels.

In contrast, AEC exhibited a different pattern of changes (Figure 2B). At baseline, strong parieto-occipital connectivity was also present, but long-range connections between frontal and parieto-occipital regions were relatively weak. Unlike wPLI, AEC did not decrease in the posterior parieto-occipital connections during moderate sedation; instead, it showed further enhancement compared to mild sedation, accompanied by widespread whole-brain coupling increases, particularly in frontal, temporal, and frontotemporal connections. During recovery, AEC returned to near-baseline levels.

Taken together, these results indicate that wPLI and AEC exhibit fundamentally different response profiles during propofol-induced sedation, confirming that the two metrics reflect distinct aspects of neural synchronization mechanisms. Statistical confirmation of these observations, based on paired *t*-tests with FDR correction, is provided in Supplementary Figure S1.

### 3.2. Dynamic Pattern Analysis of Alpha-Band wPLI During Propofol Sedation

**Figure 3:**
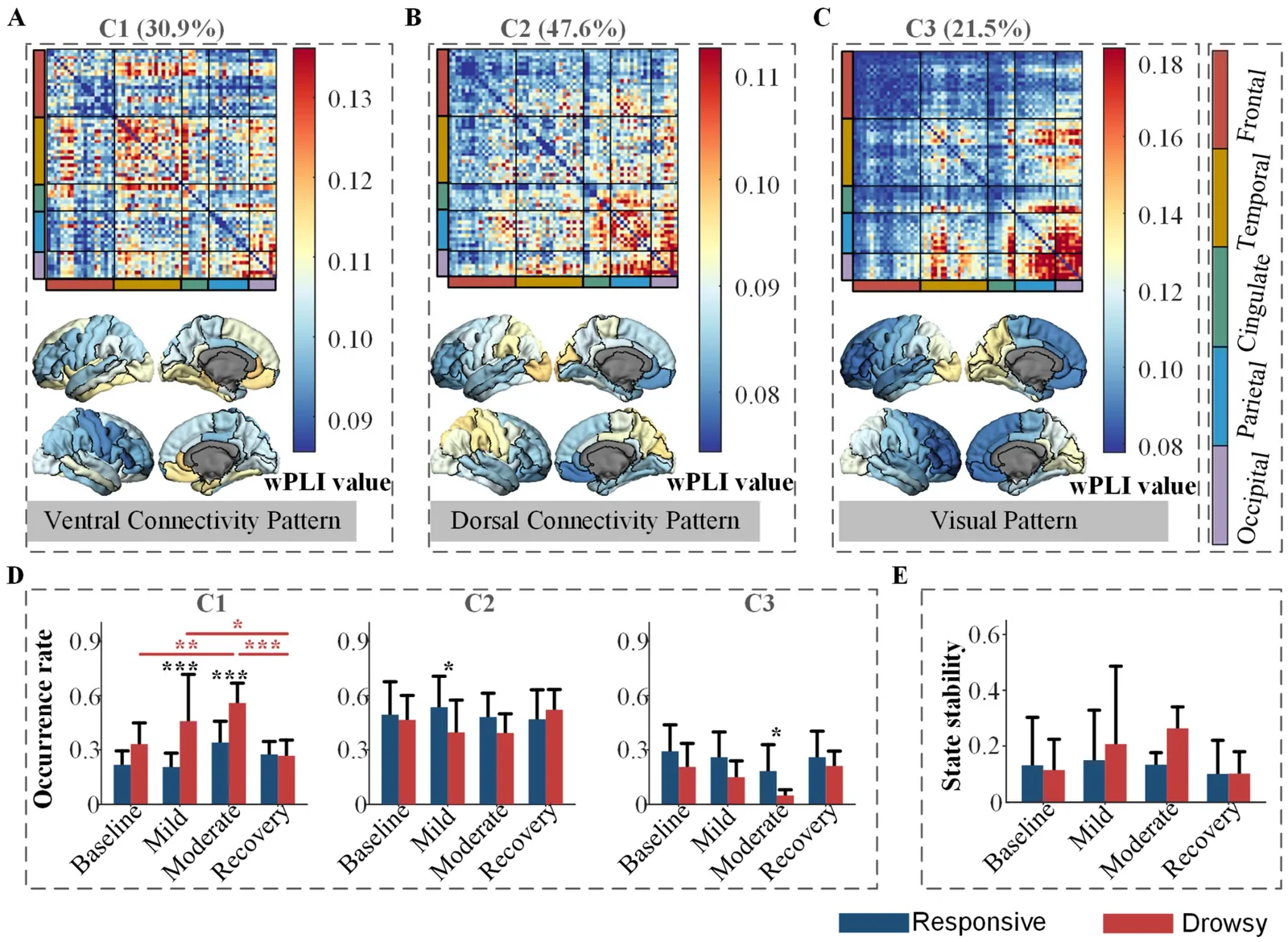
Clustering and dynamic analysis of wPLI dynamic functional connectivity: (**A**–**C**) Matrix and cortical ROI projection maps for the three wPLI dynamic functional connectivity patterns (C1, C2, C3) identified by clustering, with each ROI color representing the mean wPLI connection strength between that node and all other nodes. (**D**) Bar plots showing the occurrence rates of the three connectivity states. Blue bars represent the responsive group, and red bars represent the drowsy group. Black asterisks indicate significant differences between the two groups; red asterisks indicate significant differences across stages within the drowsy group. * *p* < 0.05, ** *p* < 0.01, *** *p* < 0.001. (**E**) Bar plots showing state stability, reflecting the mean duration of each state, with group comparisons displayed. Blue bars represent the responsive group, and red bars represent the drowsy group.

Clustering analysis was performed on the dynamic alpha-band wPLI functional connectivity matrices, which identified three connectivity patterns with distinct spatial distribution characteristics (Figure 3). C1 (Figure 3A) was the ventral connectivity pattern (accounting for 30.9% of all windows), predominantly involving connections of the visual ventral pathway and associated limbic system, with core regions including the lateral and medial orbitofrontal cortices, temporal pole, entorhinal cortex, parahippocampal gyrus, fusiform gyrus, and rostral anterior cingulate cortex. C2 (Figure 3B) was the dorsal connectivity pattern (47.6%), primarily reflecting the visual dorsal pathway and sensorimotor integration network, with core regions including the superior parietal lobule, inferior parietal lobule, supramarginal gyrus, precuneus, precentral gyrus, and paracentral lobule. C3 (Figure 3C) was the visual integration pattern (21.5%), characterized by occipital internal connections and connections from the occipital to parietal and ventral temporal regions, with core regions including the pericalcarine cortex, cuneus, lingual gyrus, and lateral occipital cortex, extending to the precuneus, isthmus cingulate, and fusiform gyrus, thus forming an integrated system from primary visual processing to higher-order visual association areas.

To investigate the differential expression of the three patterns during consciousness transitions, a linear mixed-effects model was constructed with state occurrence rate as the dependent variable. Fixed effects included stage (four levels), group (two levels), pattern type (three levels), and their interactions, with participants as random intercepts. The analysis revealed three significant effects. First, a significant pattern-by-stage interaction (*F* (6, 216) = 5.63, *p* < 0.001). Second, a significant stage-by-group interaction (*F* (2, 216) = 16.33, *p* < 0.001). Third, a significant three-way interaction (*F* (6, 216) = 2.79, *p* = 0.012). Accordingly, we proceeded with simple effects analyses, and multiple comparisons were corrected using the Tukey HSD method.

Group comparisons between the responsive and drowsy groups at each stage were first conducted (Figure 3D). During mild sedation, the responsive group showed significantly lower C1 occurrence than the drowsy group (*p* < 0.001, *d* = −1.876) and significantly higher C2 occurrence (*p* < 0.05, *d* = 1.054). During moderate sedation, the responsive group continued to show significantly lower C1 occurrence than the drowsy group (*p* < 0.001, *d* = −1.639), while exhibiting significantly higher C3 occurrence (*p* < 0.05, *d* = 0.994). No significant group differences were found for any pattern at baseline or recovery.

Subsequently, within-group comparisons across stages were performed separately for each group (Figure 3D). In the responsive group, none of the three patterns showed significant differences across stages. In the drowsy group, C1 occurrence was significantly elevated at moderate sedation relative to baseline (*p* < 0.01, *d* = −1.703). No significant stage differences were observed for C2 or C3 in either group.

The statistical results for state stability (Figure 3D) showed that neither the main effects nor the interaction of stage and group reached significance (*p* > 0.05).

### 3.3. Dynamic Pattern Analysis of Alpha-Band AEC During Propofol Sedation

**Figure 4:**
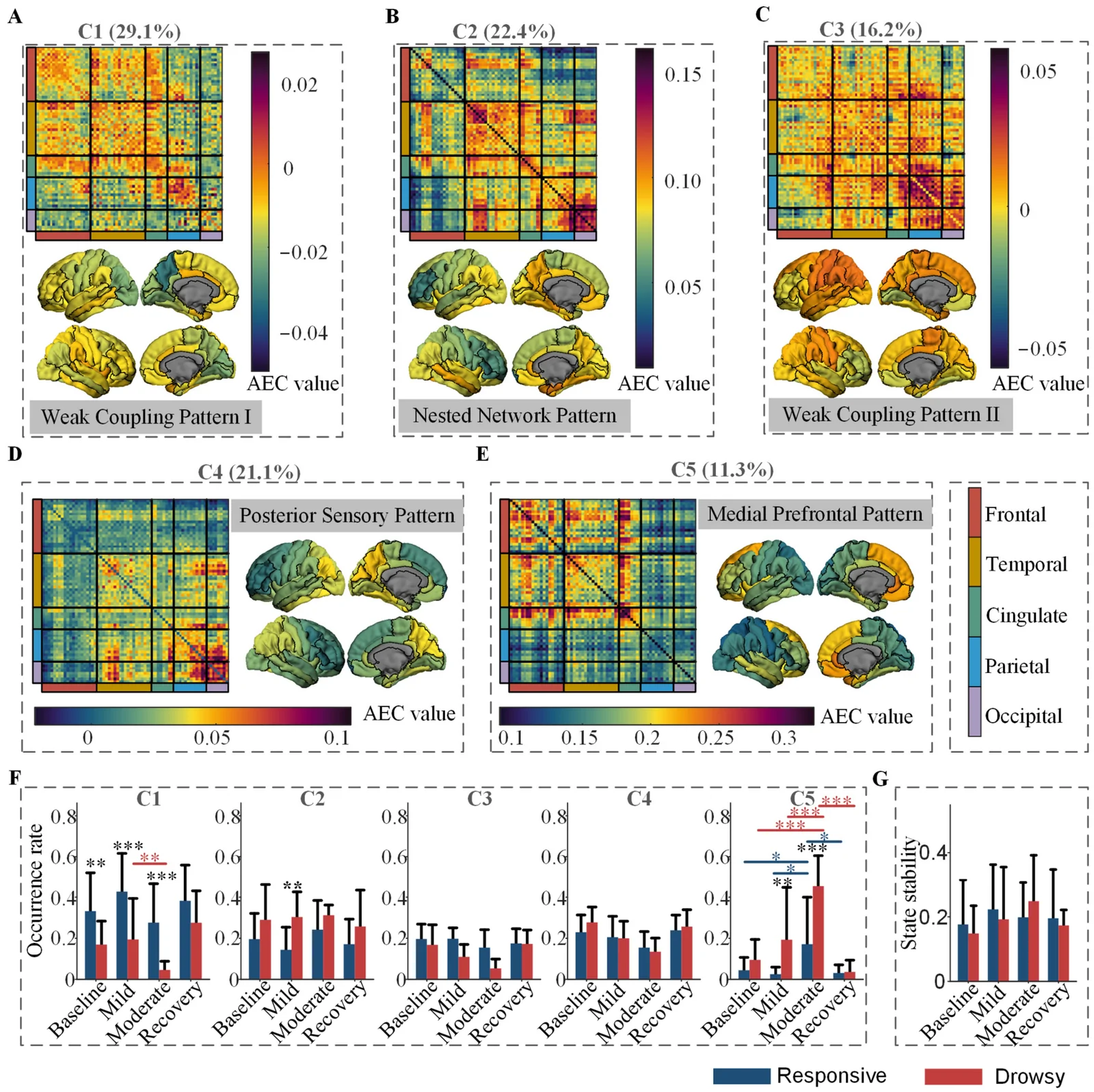
Clustering and dynamic analysis of AEC dynamic functional connectivity: (**A**–**E**) Matrix and cortical ROI projection maps for the five AEC dynamic functional connectivity patterns (C1, C2, C3, C4, and C5) identified by clustering, with each ROI color representing the mean AEC connection strength between that node and all other nodes. (**F**) Bar plots showing the occurrence rates of the five connectivity states. Blue bars represent the responsive group, and red bars represent the drowsy group. Black asterisks indicate significant differences between the two groups; blue asterisks indicate significant differences across stages within the responsive group; red asterisks indicate significant differences across stages within the drowsy group. * *p* < 0.05, ** *p* < 0.01, *** *p* < 0.001. (**G**) Bar plots showing state stability, reflecting the mean duration of each state, with group comparisons displayed. Blue bars represent the responsive group, and red bars represent the drowsy group.

Clustering analysis was performed on the dynamic alpha-band AEC functional connectivity matrices, which identified five patterns with distinct spatial distributions and connection strengths (Figure 4). C1 (Figure 4A) was a weak coupling pattern (accounting for 29.1%), characterized by generally low connectivity levels with pronounced negative connections between frontal and parieto-occipital regions. C2 (Figure 4B) was a nested network pattern (22.4%), in which alpha synchrony emerged simultaneously within the medial prefrontal cortex and within posterior sensory cortices, with the two networks interwoven to form a whole-brain functional connectivity pattern. C3 (Figure 4C) was also a weak coupling pattern (16.2%), with generally weak whole-brain connectivity, though connections within the parietal lobe and between parietal and temporo-occipital regions were relatively stronger, representing a relatively preserved connectivity hub against a background of global low connectivity. C4 (Figure 4D) was a posterior sensory pattern (21.1%), characterized by enhanced alpha activity and increased internal connectivity in posterior sensory cortices, including the parietal, occipital, and ventral temporal regions. C5 (Figure 4E) was a medial prefrontal pattern (11.3%). This pattern predominantly manifested as enhanced alpha synchrony in anterior limbic–prefrontal regions, including the medial orbitofrontal cortex, anterior cingulate cortex, and superior frontal gyrus.

To investigate the differential expression of the five patterns during consciousness transitions, a linear mixed-effects model was constructed with state occurrence rate as the dependent variable. Fixed effects included stage (four levels), group (two levels), pattern type (five levels), and their interactions, with participants as random intercepts. The results revealed a significant pattern-by-stage interaction (*F* (12, 360) = 7.65, *p* < 0.001) and a significant pattern-by-group interaction (*F* (4, 360) = 18.72, *p* < 0.001), whereas the three-way interaction did not reach significance (*F* (12, 360) = 1.77, *p* = 0.052). Given the significant two-way interaction between pattern and stage, we proceeded with simple effects analyses, with multiple comparisons corrected using the Tukey HSD method.

Group comparisons between the responsive and drowsy groups at each stage were first conducted (Figure 4F). At baseline, only C1 showed a significant difference between the two groups (*p* < 0.01, *d* = 1.315), with the responsive group showing a higher occurrence than the drowsy group. During mild sedation, significant group differences were observed for C1 (*p* < 0.001, *d* = 1.875), C2 (*p* < 0.01, *d* = −1.285), and C5 (*p* < 0.01, *d* = −1.353), with the responsive group showing higher C1 occurrence and lower C2 and C5 occurrence compared to the drowsy group. During moderate sedation, significant group differences were found for C1 (*p* < 0.001, *d* = 1.861) and C5 (*p* < 0.001, *d* = −2.283), with the responsive group showing higher C1 occurrence and lower C5 occurrence than the drowsy group. No significant group differences were found for any pattern at recovery.

Subsequently, within-group comparisons across stages were performed separately for each group (Figure 4F). In the responsive group, C1 showed a significant difference between mild and moderate sedation (*p* < 0.05, *d* = 1.214), with lower occurrence at moderate sedation relative to mild sedation; C5 was significantly elevated at moderate sedation compared to baseline (*p* < 0.05, *d* = −1.022) and to mild sedation (*p* < 0.05, *d* = −1.179) and returned to significantly lower levels at recovery relative to moderate sedation (*p* < 0.05, *d* = 1.131). In the drowsy group, C1 was significantly elevated at recovery compared to moderate sedation (*p* < 0.01, *d* = −1.855); C5 was significantly elevated at moderate sedation compared to baseline (*p* < 0.001, *d* = −2.900) and to mild sedation (*p* < 0.001, *d* = −2.109) and significantly decreased at recovery relative to moderate sedation (*p* < 0.001, *d* = 3.371), with no significant difference between recovery and baseline (*p* > 0.05).

The statistical results for state stability (Figure 4G) showed that neither the main effects nor the interaction of stage and group reached significance (*p* > 0.05).

### 3.4. Changes in Joint Distribution of Alpha-Band wPLI and AEC Dynamic Patterns During Propofol Sedation

**Figure 5:**
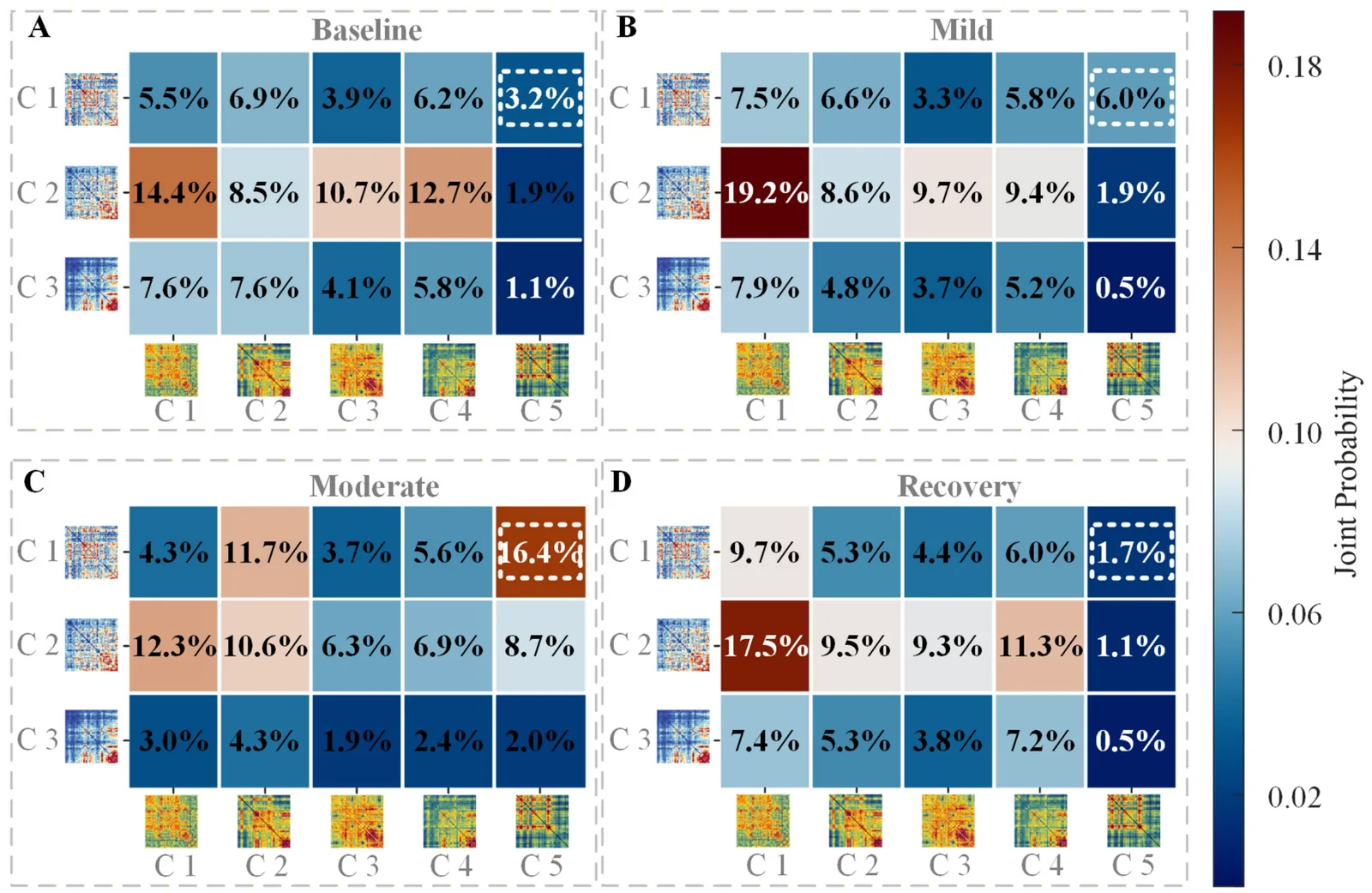
Joint probability distributions of wPLI and AEC dynamic functional connectivity clustering patterns: (**A**–**D**) Joint probability matrices for the four stages: baseline, mild sedation, moderate sedation, and recovery, respectively. The vertical axis (C1–C3) represents the three wPLI dynamic functional connectivity clustering patterns, and the horizontal axis (C1–C5) represents the five AEC dynamic functional connectivity clustering patterns. The color intensity of each cell in the matrix indicates the joint probability of the corresponding wPLI-AEC pattern combination, with the percentage value annotated within each cell. Warm colors (red) indicate high joint probabilities, whereas cool colors (blue) indicate low joint probabilities. The small panels to the left of each row and below each column show the functional connectivity matrices of the corresponding wPLI and AEC clustering patterns, respectively. The white dashed boxes in the figure indicate regions of interest.

To examine the co-occurrence relationship between wPLI and AEC dynamic patterns, we performed a joint distribution analysis on the clustering results of the two metrics, calculating the co-occurrence probabilities of the three wPLI patterns and five AEC patterns across different stages (Figure 5). The results showed that the joint distribution probability of the wPLI C1 pattern and the AEC C5 pattern increased markedly from baseline to mild-to-moderate sedation, suggesting a potential synergistic enhancement of this combined pattern during sedation.

## 4. Discussion

The present study aimed to examine whether phase synchrony and amplitude envelope coupling in the alpha band follow the same principles of dynamic reorganization during propofol sedation. By performing clustering analyses on dynamic functional connectivity for wPLI and AEC separately, we found that the two metrics exhibited spatially distinct patterns of dynamic transitions during sedation. Specifically, the occurrence rates of both the wPLI ventral connectivity pattern and the AEC medial prefrontal pattern increased with deepening sedation and returned toward baseline during recovery. This effect was more pronounced in the drowsy group. Collectively, these findings suggest that propofol-induced alterations in consciousness do not arise from linear attenuation along a single dimension of neural synchrony, but rather from differential reorganization of phase- and amplitude-based functional connectivity across spatial configurations and temporal dynamics.

From a neurophysiological perspective, wPLI measures precise temporal synchrony between brain regions, relying on the accurate temporal alignment of pyramidal neuron firing. Propofol, via GABA_A receptor-mediated inhibition, prolongs inhibitory postsynaptic decay, thereby impairing precise phase locking between distant regions—consistent with prior reports of propofol-induced loss of posterior alpha coherence and attenuated frontoparietal coupling [37,38]. Notably, in the present study, the decrease in wPLI connectivity strength was predominantly concentrated in posterior parieto-occipital regions, suggesting a selective disruption of fine-grained temporal communication along visual processing pathways [39].

In contrast, AEC reflects the co-fluctuation of population-level neuronal excitability on a slower timescale [17]. Propofol-enhanced GABAergic inhibition can drive thalamocortical amplitude synchronization via rhythmic oscillations in thalamic circuitry [12,38]. We therefore speculate that the sedation-related increase in AEC may reflect an “idling” mode of the cortex under deep inhibition. This mode could potentially be characterized by increased energy coupling alongside reduced information capacity [8]. This interpretation aligns with evidence showing that enhanced amplitude envelope coupling during anesthesia is accompanied by loss of information diversity [40] and that excessive rigidity of dynamic functional connectivity correlates with reduced consciousness levels [41].

At the level of dynamic patterns, the ventral connectivity pattern identified by wPLI increased significantly in the sleepiness group during sedation [42], whereas the dorsal connectivity pattern showed no corresponding change, suggesting that the ventral pattern has higher sensitivity to the sedative effects of propofol. The ventral connectivity pattern extends along the temporal lobe to the medial temporal lobe and orbitofrontal cortex. The brain regions involved in this pattern have extensive and close anatomical connections with the limbic system [43] and are deeply integrated into the ‘sensory–limbic’ network. This tight integration with the limbic system likely constitutes the structural basis for the higher sensitivity of the ventral pathway to propofol.

For AEC dynamics, the most prominent change was the dose-dependent elevation of the medial prefrontal pattern. The medial prefrontal cortex is a core hub of the default mode network [44], and propofol is known to enhance alpha power in this region [45]. Our dynamic analysis further demonstrated that this local power enhancement extended to a widespread increase in frontal amplitude envelope coupling [12]. Group comparisons revealed that the drowsy group exhibited greater elevation of this pattern, suggesting a dose-response relationship between prefrontal amplitude synchronization and the loss of behavioral responsiveness.

The increased joint probability of the wPLI ventral connectivity pattern and the AEC medial prefrontal pattern during sedation further indicates that these two patterns tend to co-occur under sedation. This finding is consistent with a recent theoretical framework in which consciousness states are characterized by multidimensional brain network configurations [46], suggesting that anesthetic-induced consciousness alteration is not a global inhibition but rather a reorganization of specific functional configurations.

This study has several limitations. First, our analysis was confined to the alpha band, leaving slower oscillations such as delta and theta unexamined. Second, the spatial resolution of source imaging precluded direct investigation of subcortical structures, including the thalamus. Third, the relatively small sample size, particularly the drowsy subgroup of only seven participants, may have limited statistical power. Consequently, the behavioral differences and recovery-related effects observed in the drowsy group should be interpreted with caution, and future studies with larger samples are warranted to confirm these findings. Fourth, the recovery-stage recordings were acquired shortly after cessation of propofol administration and may not reflect full neurophysiological recovery, particularly in the drowsy subgroup; future studies should include extended recovery monitoring to capture the complete trajectory of functional state restoration. Despite these limitations, by concurrently analyzing dynamic functional connectivity from both phase and amplitude dimensions, this study provides multidimensional evidence for the differential reorganization of brain networks during propofol sedation. These findings support the view that consciousness states are characterized by complex spatiotemporal configurations of multiple neural synchrony patterns and lay a preliminary foundation for developing consciousness monitoring approaches grounded in multidimensional connectivity dynamics. Future studies with larger samples, incorporating cross-frequency analyses and causal interventions, are warranted to further validate the reorganization mechanisms identified here.

## 5. Conclusions

This study simultaneously examined dynamic functional connectivity based on wPLI and AEC, revealing differential reorganization of alpha-band brain networks during propofol sedation. The elevated occurrence rates of both the wPLI ventral connectivity pattern and the AEC medial prefrontal pattern with deepening sedation jointly constituted a multidimensional network signature of propofol-induced consciousness transition. These findings suggest that dynamic fluctuations in consciousness levels do not arise from monotonic changes in a single neural process, but rather from the differential reorganization of distinct types of neural synchrony across spatial configurations and temporal dynamics. This multidimensional dynamic perspective has implications for understanding anesthetic mechanisms, developing consciousness monitoring indicators, and exploring the neural basis of disorders of consciousness.

## Figures and Tables

**Table 1:** Desikan–Killiany atlas parcellation.

Region	ROI Names
Frontal	frontalpole; parsorbitalis; lateralorbitofrontal; medialorbitofrontal; rostralmiddlefrontal; parstriangularis; parsopercularis; superiorfrontal; caudalmiddlefrontal; precentral
Temporal	temporalpole; entorhinal; parahippocampal; fusiform; superiortemporal; middletemporal; inferiortemporal; transversetemporal; bankssts; insula
Cingulate	rostralanteriorcingulate; caudalanteriorcingulate; posteriorcingulate; isthmuscingulate
Parietal	paracentral; postcentral; supramarginal; superiorparietal; inferiorparietal; precuneus
Occipital	lingual; pericalcarine; cuneus; lateraloccipital

## Data Availability

The open-source data can be accessed through the Repository URL https://www.repository.cam.ac.uk/handle/1810/252736 (accessed on 12 June 2026).

## Supplementary Materials

The following supporting information can be downloaded at: https://www.mdpi.com/article/10.3390/brainsci16080866/s1, Figure S1. Pairwise *t* test results of static functional connectivity matrices for wPLI and AEC; Figure S2. Clustering solution determination and robustness validation for wPLI dynamic connectivity; Figure S3. Clustering solution determination and robustness validation for AEC dynamic connectivity.
