# Supplementary material for: Differential Dynamic Reorganization of Functional Connectivity Based on Phase Synchrony and Amplitude Envelope Coupling During Propofol Sedation
Source: Brain Sci. 2026 Aug 16;16(8):866. doi: 10.3390/brainsci16080866 (PMC13510883; doi:10.3390/brainsci16080866)
Supplement: Supplementary file 1 [file brainsci-16-00866-s001.zip › brainsci-4439063-supplementary.pdf]

Supplementary Material

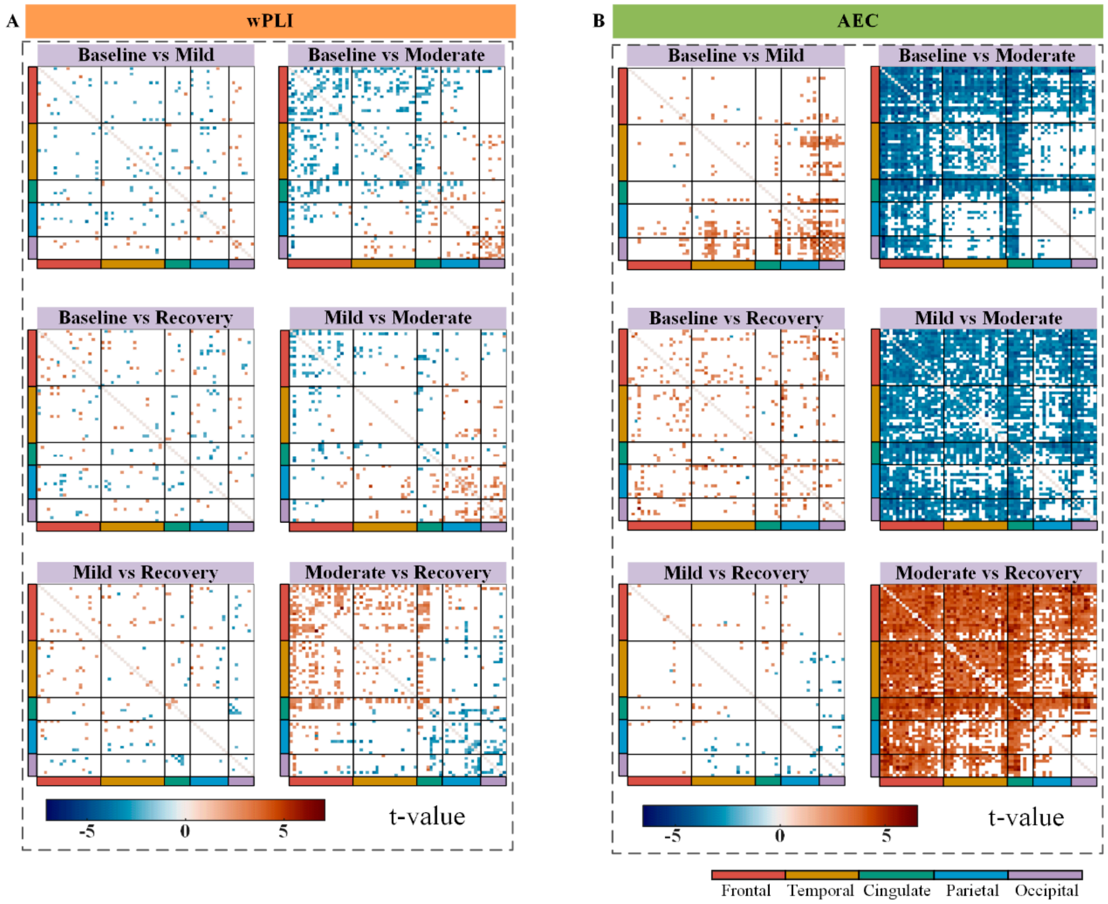

Figure S1. Pairwise t-test results of static functional connectivity matrices for wPLI and AEC. (A) wPLI results; (B) AEC results. Heatmaps display pairwise comparisons of functional connectivity strength across four anesthesia states (Baseline, Light sedation, Moderate sedation, and Recovery), resulting in 6 contrasts in total. Only connections surviving FDR correction (Benjamini-Hochberg procedure, $q < 0.05$) are shown; non-significant regions are filled in white. Red indicates that connectivity in the former state (State 1) is significantly higher than in the latter (State 2), while blue indicates the opposite; color intensity reflects the magnitude of the t-value. All subplots share a unified color scale to facilitate direct comparison across different contrasts.

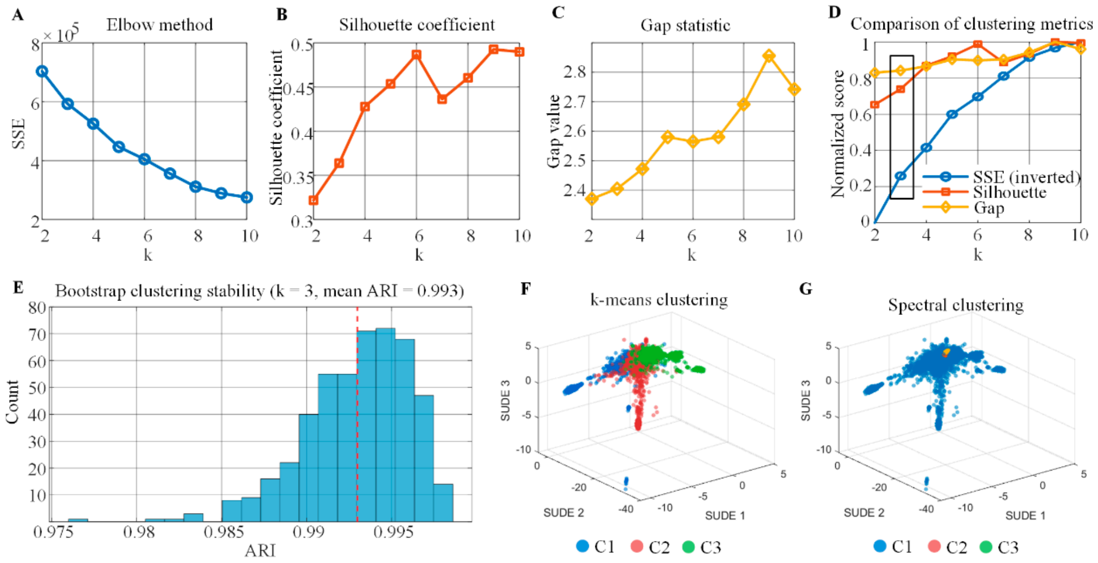

Figure S2. Clustering solution determination and robustness validation for wPLI dynamic connectivity. (A) Elbow method based on within-cluster sum of squared errors (SSE), where the optimal k is identified at the inflection point of the curve; (B) average Silhouette coefficient, with higher values indicating better clustering quality; (C) Gap statistic computed with 50 reference datasets (PCA reference distribution), where the optimal k corresponds to the maximum Gap value; (D) normalized comparison of all metrics (all metrics scaled to [0, 1], with SSE inverted so that higher scores indicate better performance). Based on the integrated assessment in (A)–(D), the optimal number of clusters for the AEC data was determined as k = 3. (E) Bootstrap clustering stability assessment (k = 3, B = 500). The histogram shows the distribution of Adjusted Rand Index (ARI) values obtained from 500 Bootstrap resampling iterations, each comparing the resampled clustering solution with the original clustering solution. The red dashed line indicates the mean ARI (0.993), substantially exceeding the commonly accepted stability threshold of 0.8, demonstrating high reproducibility and robustness of the clustering solution. (F–G) Visualization of K-means and spectral clustering results in the first three dimensions of the SUDE embedding space. (F) K-means clustering result (k = 3), with different colors representing distinct state clusters; (G) spectral clustering result (k = 3), with the same color scheme as in (F).

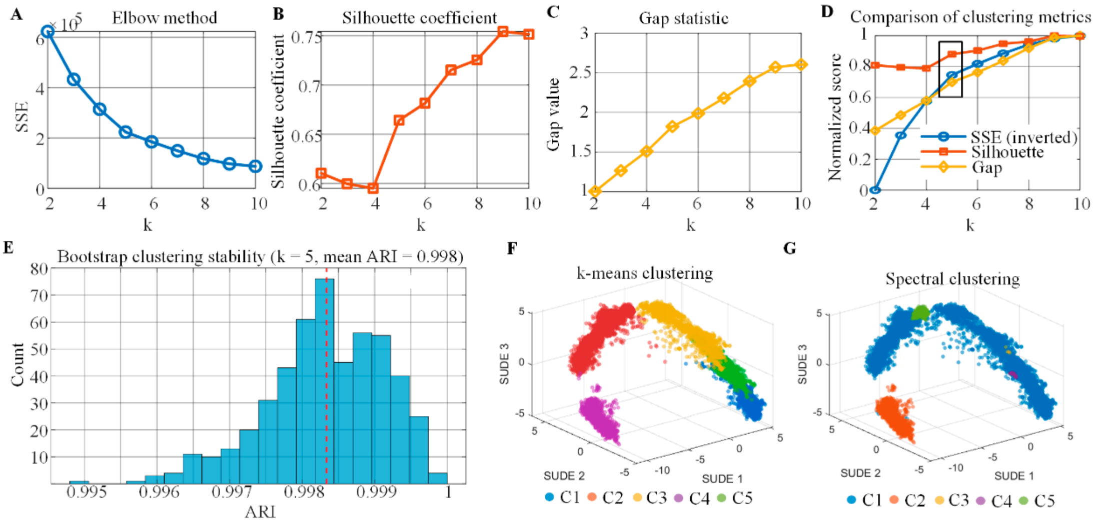

Figure S3. Clustering solution determination and robustness validation for AEC dynamic connectivity. (A) Elbow method based on within-cluster sum of squared errors (SSE), where the optimal k is identified at the inflection point of the curve; (B) average Silhouette coefficient, with higher values indicating better clustering quality; (C) Gap statistic (mean ± SE) computed with 50 reference datasets (PCA reference distribution), where the optimal k corresponds to the maximum Gap value; (D) normalized comparison of all metrics (all metrics scaled to [0, 1], with SSE inverted so that higher scores indicate better performance). Based on the integrated assessment in (A)–(D), the optimal number of clusters for the AEC data was determined as k = 5. (E) Bootstrap clustering stability assessment (k = 5, B = 500). The histogram shows the distribution of Adjusted Rand Index (ARI) values obtained from 500 Bootstrap resampling iterations, each comparing the resampled clustering solution with the original clustering solution. The red dashed line indicates the mean ARI (0.998), substantially exceeding the commonly accepted stability threshold of 0.8, demonstrating high reproducibility and robustness of the clustering solution. (F–G) Visualization of K-means and spectral clustering results in the first three dimensions of the SUDE embedding space. (F) K-means clustering result (k = 5), with different colors representing distinct state clusters; (G) spectral clustering result (k = 5), with the same color scheme as in (F).
